# Cardiopoietic stem cell therapy restores infarction-altered cardiac proteome

**DOI:** 10.1038/s41536-020-0091-6

**Published:** 2020-03-12

**Authors:** D. Kent Arrell, Christian S. Rosenow, Satsuki Yamada, Atta Behfar, Andre Terzic

**Affiliations:** 1 Center for Regenerative Medicine, Mayo Clinic, Rochester, MN USA; 2 Marriott Heart Disease Research Program, Mayo Clinic, Rochester, MN USA; 3 Van Cleve Cardiac Regenerative Medicine Program, Mayo Clinic, Rochester, MN USA; 4 Department of Cardiovascular Medicine, Mayo Clinic, Rochester, MN USA; 5 Department of Molecular Pharmacology & Experimental Therapeutics, Mayo Clinic, Rochester, MN USA; 6 Division of Geriatric Medicine & Gerontology, Department of Medicine, Mayo Clinic, Rochester, MN USA; 7 Department of Physiology and Biomedical Engineering, Mayo Clinic, Rochester, MN USA; 8 Department of Medical Genetics, Mayo Clinic, Rochester, MN USA

**Keywords:** Regeneration, Heart failure

## Abstract

Cardiopoietic stem cells have reached advanced clinical testing for ischemic heart failure. To profile their molecular influence on recipient hearts, systems proteomics was here applied in a chronic model of infarction randomized with and without human cardiopoietic stem cell treatment. Multidimensional label-free tandem mass spectrometry resolved and quantified 3987 proteins constituting the cardiac proteome. Infarction altered 450 proteins, reduced to 283 by stem cell treatment. Notably, cell therapy non-stochastically reversed a majority of infarction-provoked changes, remediating 85% of disease-affected protein clusters. Pathway and network analysis decoded functional reorganization, distinguished by prioritization of vasculogenesis, cardiac development, organ regeneration, and differentiation. Subproteome restoration nullified adverse ischemic effects, validated by echo-/electro-cardiographic documentation of improved cardiac chamber size, reduced QT prolongation and augmented ejection fraction post-cell therapy. Collectively, cardiopoietic stem cell intervention transitioned infarcted hearts from a cardiomyopathic trajectory towards pre-disease. Systems proteomics thus offers utility to delineate and interpret complex molecular regenerative outcomes.

## Introduction

Cardiopoiesis leverages natural developmental cues to impart lineage engagement for enhanced cardioreparative outcome^1–3^. Applied to adult stem cells, recombinant growth factor-induced cardiopoiesis disrupts latent plasticity to prime cardiovasculogenesis while maintaining a proliferative state^2,3^. Supported by preclinical studies^4,5^, cardiopoietic stem (CP) cell-based therapy for heart failure is undergoing clinical evaluation^6,7^. While global readouts of functional and structural safety and efficacy have been the focus of exploration to date^8,9^, delineation of the molecular impact of CP cells upon the recipient heart has yet to be charted.

Systems proteomics offers a precision means of resolving the complexity of stem cell therapy^10–12^. Proteomic capabilities now resolve and quantify thousands of proteins, providing throughput similar to next-generation RNA sequencing^13^. With proteins executing the majority of cellular structural and functional roles^14^, proteome analytics obviates misinterpretation due to discrepancies between transcript and protein readouts arising from post-transcriptional processing and splicing, and differing mRNA versus corresponding protein isoform half-lives. Indeed, use of proteomics-based assessment has proven increasingly valuable for comprehending cardiovascular biology in health and disease^15–18^.

In parallel, advances in systems biology provide inclusive, unbiased strategies to address the magnitude of high throughput datasets, where interpretation on a protein by protein basis is intractable^19,20^. Selective focus on a handful of proteins, such as expression extremes or those associated with a particular pathology, simplifies analysis but may lead to selection bias, and invariably accrue data loss including information germane to a comprehensive interpretation^21^. Modern systems biology approaches offer the capacity to reduce data dimensionality and enable functional prioritization, without the need to resort to subjective data exclusion^19–22^.

Accordingly, proteomic profiling along with multi-parametric systems interrogation was here applied to characterize cardiac molecular maladaptation to ischemic cardiomyopathy, and delineate the response of diseased hearts to CP cell treatment. To this end, cells were lineage guided from human bone marrow-derived mesenchymal stem cells (MSCs), consistent with clinical trial cell sourcing^6,7^. Therapeutic application of human CP cells in a xenograft model of ischemic cardiomyopathy enabled whole ventricle evaluation unachievable from clinical trial participants. This integrative approach resolved widespread proteome remodeling within the infarcted substrate, and captured a non-random reversal of these disease-perturbed derangements following stem cell treatment.

## Results

### Cardiac proteome resolution

Sequential extraction of cardiac ventricular tissue, followed by quantitative multi-dimensional label-free tandem mass spectrometry, yielded 47,220 peptides. Accounting for 133 reverse sequence and 207 contaminant peptides, the resolved proteome included 46,880 total peptides (Supplementary Data Set 1) allocated to 3987 proteins (Supplementary Data Set 2), for a mean of 11.8 and median of 7 peptides per protein. The proportion of proteins detected ranged from 99.6–99.8% across control (Ctrl), infarcted without (MI), or infarcted with CP cell therapy (MI + CP) groups (*n* = 4 per group; Supplementary Data Set 2). Consistent inter-group protein spectral intensities (Supplementary Data Fig. 1A) with narrow intra-group abundance variance (Supplementary Data Fig. 1B) enabled inclusive, reliable elucidation of proteome complexity, and differential abundance assessment in response to disease and treatment.

### Infarcted proteome is responsive to stem cell therapy

**Fig. 1:**
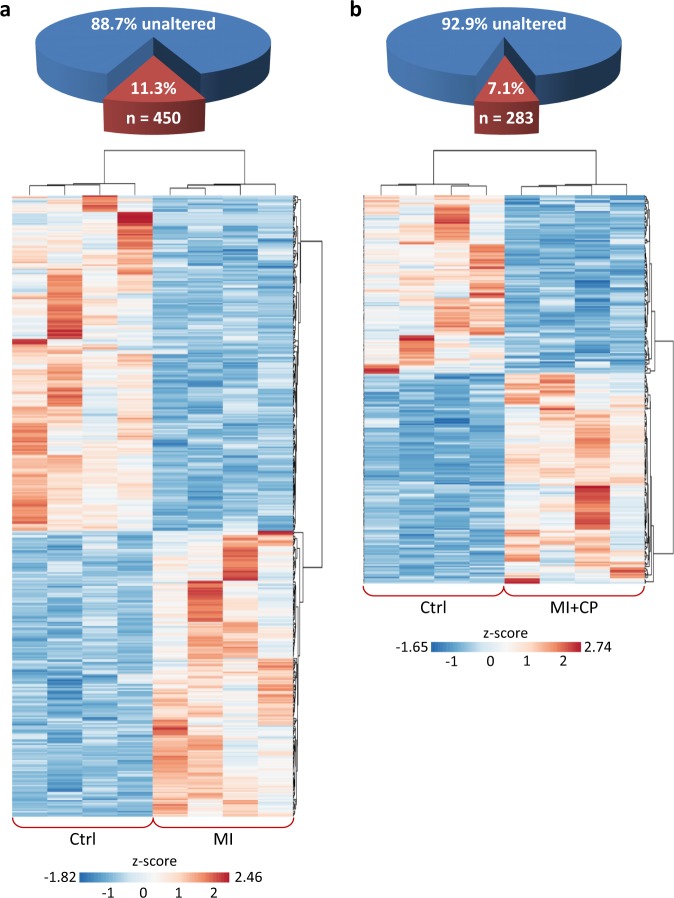
Cardiopoietic cell therapy reduces infarction altered cardiac proteome. **a** Of the 3987 identified ventricular proteins, 450 (11.3%) differed significantly between Ctrl and MI (two-fold change, *p* < 0.01), with *z*-score transformed agglomerative heatmap showing 243 downregulated (blue) and 207 upregulated (red) proteins in infarcted hearts. **b** CP cell treatment reduced infarction-induced change to 283 differences from Ctrl (7.1% of total), with heatmap detailing 130 down (blue) and 153 up (red). Ctrl control, MI myocardial infarction, MI + CP MI plus cardiopoietic cell therapy.

Over 11% of the ventricular proteome was significantly altered 8 weeks post-infarction (Fig. 1a upper), with 207 proteins upregulated and 243 downregulated compared to pre-infarction levels (Fig. 1a lower; >2-fold, *p* < 0.01). Four weeks of CP cell therapy diminished extent of change to 7% (Fig. 1b upper), with 153 proteins increased and 130 decreased (Fig. 1b lower). Cell therapy eliminated 37% of infarction-induced proteome changes, with downregulated proteins reduced to 54% (130/243), and upregulated proteins to 74% (153/207) of infarction levels. Thus, chronic ischemia significantly alters the cardiac proteome, yet the infarcted heart remains malleable and responsive to CP cell treatment.

### Cell therapy non-randomly reverses infarcted proteome changes

**Fig. 2:**
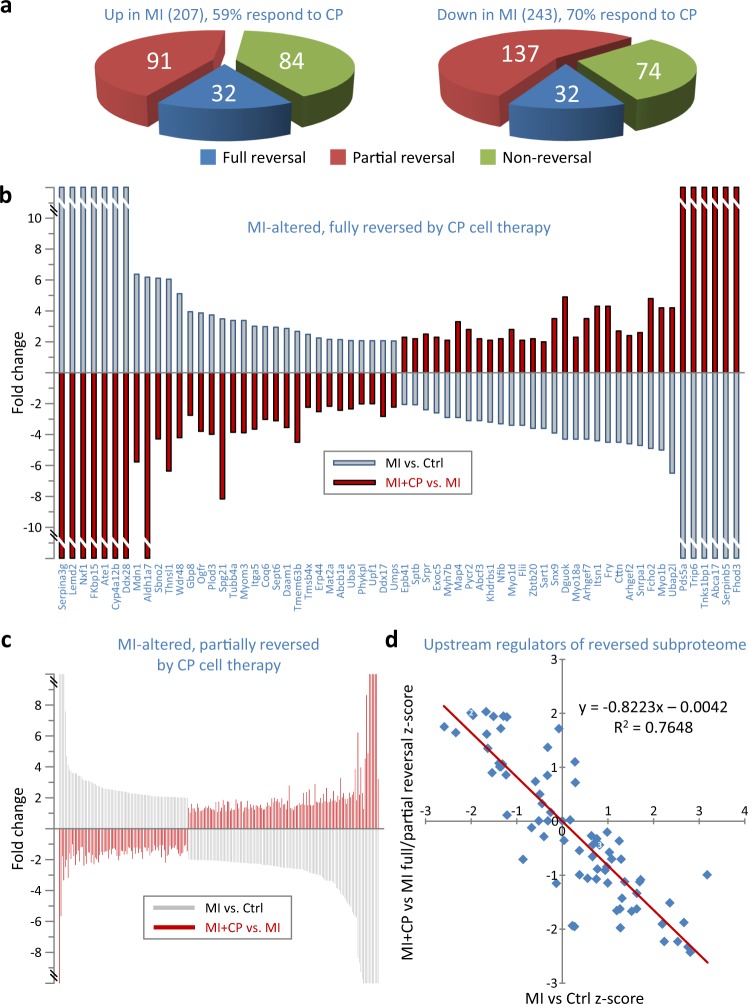
Reversal of infarcted proteome and predicted upstream regulators. **a** A majority of proteins altered by MI responded to cardiopoietic (CP) cell therapy (*n* = 292, 64.9%), either by full (*n* = 64, 14.2%, blue) or partial (*n* = 228, 50.7%, red) reversal, whereas 158 proteins (35.1%, green) did not. A larger proportion of downregulated (70%) than upregulated (59%) proteins were responsive to therapy. **b** Full reversal, following cell treatment, occurred for 32 proteins upregulated and 32 downregulated by infarction. Proteins altered in MI vs. Ctrl (gray bars) were considered fully reversed when altered in the opposite direction in MI + CP vs. MI (red bars) such that MI + CP did not differ from Ctrl. **c** Partial reversal occurred for 91 proteins upregulated and 137 downregulated by infarction. Proteins considered partially reversed were altered in the opposite direction in MI + CP (versus MI), and either without reaching significance versus MI and not differing from Ctrl; or significantly differing from both Ctrl and MI. **d** Reversed subproteome (*n* = 292 proteins) interpretation by pathway analysis identified 78 upstream regulators possessing *z*-scores for both infarction-induced (MI versus Ctrl) and reversed (MI + CP versus MI) proteome changes, with large *z*-scores predictive of activation (positive *z*-score) or inhibition (negative *z*-score). *z*-score inverse correlation suggested upstream regulators activated by MI were inhibited by cell therapy, and vice versa. Several cardiac and stem cell transcription factors localize to the upper left quadrant, indicating predicted inhibition in MI, with re-activation upon CP cell therapy. Ctrl control, MI myocardial infarction, MI + CP MI plus cardiopoietic cell therapy.

CP cell therapy either fully or partially reversed 65% of all proteins altered by infarction (292 of 450), including 59% that MI upregulated and 70% that MI downregulated (Fig. 2a). For 64 MI altered proteins (32 up, 32 down, Fig. 2a blue), full reversal was achieved such that they no longer differed from controls, mirroring the MI landscape with expression altered significantly and in the opposite direction (Fig. 2b). Partial reversal was attained for 228 other infarction-altered proteins (91 up, 137 down, Fig. 2a red, Fig. 2c). The majority (222/228) also no longer differed from controls, but did not reach statistical significance when compared to MI expression. For six additional proteins, MI + CP versus MI expression reversal was significant; however, these six also remained different from controls (MI + CP versus Ctrl). Pathway analysis further unmasked an inverse correlation between predicted MI- versus CP cell therapy-induced activation or inhibition of upstream regulators underlying the fully and partially reversed subproteome (Fig. 2d). Thus, CP cell therapy mediated a targeted reversal of two-thirds of the MI corrupted proteome.

### Cell therapy remediates infarction-affected protein classes

**Fig. 3:**
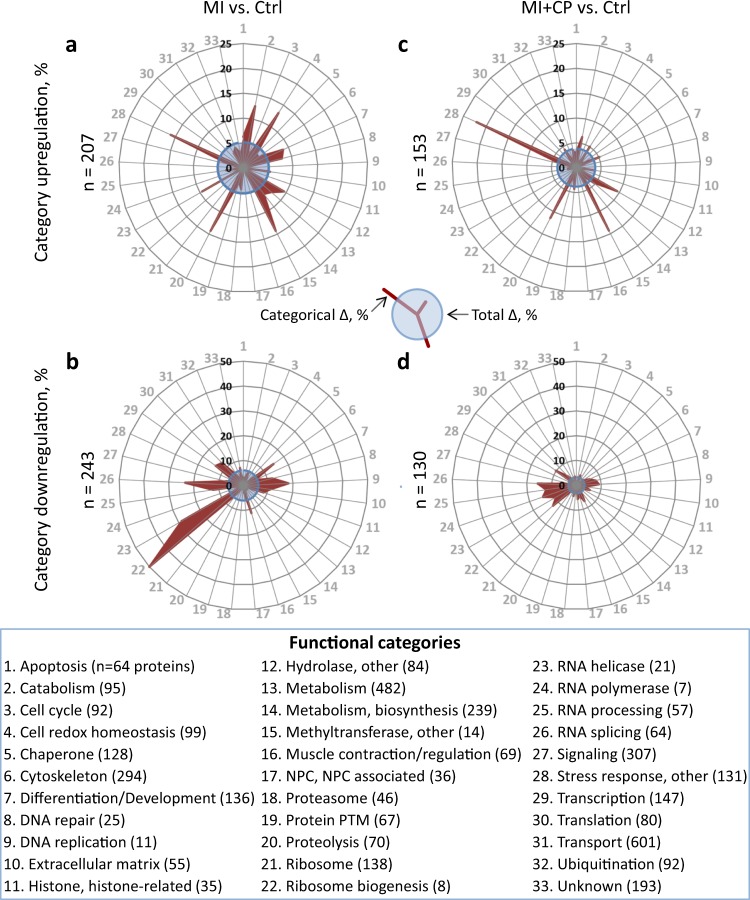
Infarction susceptible protein classes respond to cell therapy. Categorical impact was assessed for 33 functional classes into which proteins were allocated, with radial graphs plotting changes induced by myocardial infarction without (MI vs. Ctrl; upregulated in **a**, downregulated in **b**) or with cardiopoietic cell therapy (MI + CP vs. Ctrl; upregulated in **c**, downregulated in **d**). Radial displacement (red projection) indicates intra-class percentage differing from controls (Categorical Δ, %). Blue circles represent total proteome change (Total Δ, %). Proteins upregulated by infarction were reduced for most categories (26% overall) by cell therapy (**a** versus **c**), and likewise for categories downregulated by infarction (**b** versus **d**), which reduced 46.5% overall. For each graph **a**–**d**, *n* indicates number of proteins changed (>2-fold, *p* < 0.01). Functional categories are arrayed alphabetically, with identities and number of proteins listed.

Protein categorization demonstrated an extensive impact by MI, with upregulation (Fig. 3a) or downregulation (Fig. 3b) across 32 of 33 designated classes. Twelve categories overrepresented by protein upregulation, where proportion of change exceeded the 5.2% upregulated frequency of the full proteome (Fig. 3a, blue circle), included: apoptosis; catabolism; cell redox homeostasis; differentiation and development; DNA repair; hydrolases; metabolism; metabolism—biosynthesis; methyltransferases; proteolysis; RNA helicases; and stress response. A reciprocal effect was observed for 14 overrepresented and functionally downregulated clusters affected at a prevalence greater than the 6.1% downregulation frequency of the complete proteome (Fig. 3b, blue circle): cytoskeleton; DNA repair; DNA replication; extracellular matrix; histones and histone-related proteins; muscle contraction and regulation; ribosome biogenesis; RNA helicases; RNA processing; RNA splicing; signaling; transcription; translation; and proteins of unknown function. With the exception of DNA repair and RNA helicases, these downregulated and upregulated categories were mutually exclusive. Thus, chronic MI impacted the proteome across essentially all categorical domains, extensively upregulating specific protein classes while downregulating other, functionally distinct categories.

CP cell therapy blunted MI-induced change, with 54 fewer upregulated (Fig. 3c versus 3a, blue circles) and 113 fewer downregulated (Fig. 3d versus Fig. 3b, blue circles) proteins. Cell treatment widely targeted the proteome, notably reducing change for 22 of the 26 overrepresented categories reciprocally remodeled by MI (compare radial projections in Fig. 3c versus Fig. 3a and Fig. 3d versus Fig. 3b). For proteins upregulated by infarction, extent of change for 16 of 33 clusters was reduced following CP cell therapy (Fig. 3c), accounting for 70 fewer disease-affected proteins. These clusters included 9 of the 12 overrepresented upregulation categories. In parallel, 12 clusters remained unchanged, while for 5 remaining clusters 16 additional protein differences were observed. CP cell therapy resulted in a net decrease of 54 upregulated proteins (*n* = 207 in Fig. 3a versus *n* = 153 in Fig. 3c). For proteins downregulated by infarction, extent of change for 21 of 33 clusters was reduced by cell therapy (Fig. 3d), accounting for 122 fewer disease affected proteins. Among these clusters were 13 of the 14 overrepresented downregulation categories. Five clusters were unchanged, and in the seven remaining clusters nine additional protein differences were observed for a net decrease of 113 downregulated proteins (*n* = 243 in Fig. 3c versus *n* = 130 in Fig. 3d). Thus, CP cell therapy had a targeted impact spanning the spectrum and directionality of infarction-affected protein categories.

### Cell therapy reorganizes proteome neighborhood functional prioritization

**Fig. 4:**
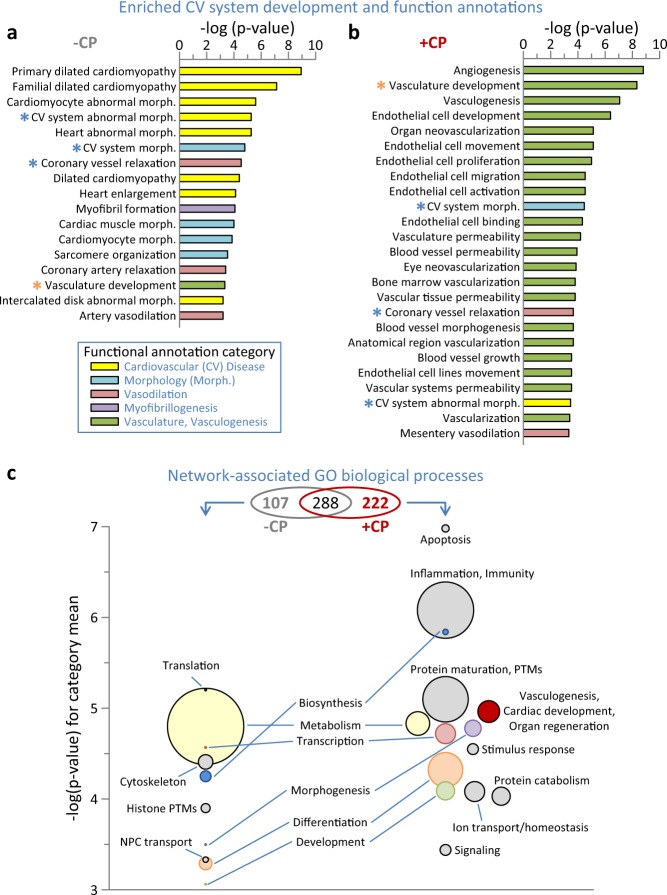
Functional enrichment at proteome and network levels indicative of cell therapy impact. **a** Screening of proteins differentially expressed by myocardial infarction (–CP) for Ingenuity Pathway Analysis (IPA) enrichment (*p* < 0.001) of cardiovascular system development and physiological functions revealed prioritization of cardiovascular disease and abnormal morphology. **b** IPA screening of the cell therapy differential proteome (+CP) indicated a transition from infarction, with numerous enriched functions prioritizing vasculogenesis and associated vascular properties. Asterisks indicate annotations common to **a** and **b**, shown in blue or orange if reduced or increased in significance, respectively, following cell therapy (+CP versus –CP). **c** Venn diagram indicates enriched (Benjamini–Hochberg corrected *p* < 0.001) Gene Ontology (GO) Biological Processes shared between or unique to the –CP and +CP networks from Supplementary Data Fig. 2. Bubble plots indicate processes unique to each network, with circle diameter proportional to intra-cluster number of annotations, and −log(*p*-value) for each circle representing the mean of all annotations represented within the cluster. Clusters present in both networks are noted between the –CP and +CP bubble plots, with matching categories shown in identical colors.

Functional enrichment analyses (FDR corrected *p* < 0.001) revealed that cardiomyopathy and associated manifestations characterized the untreated MI proteome (Fig. 4a). CP cell treatment transformed this landscape, blunting or eliminating disease and abnormal traits while enriching for vasculogenic, angiogenic, and related endothelial development and function (Fig. 4b). Integrated interactions, encompassing affected protein neighborhoods^20–22^, yielded scale-free MI-untreated and cell-treated networks comprised of 768 (Supplementary Data Fig. 2A) and 578 (Supplementary Data Fig. 2B) nodes, respectively. Network composition transitioned in response to therapy, with <50% of nodes retained post-treatment (Supplementary Data Fig. 2B inset, highlighted as yellow nodes in Supplementary Data Fig. 2A, B) unmasking distinguishing biological processes (FDR corrected *p* < 0.001) characterizing the individual networks (Fig. 4c upper, Venn diagram). Within the 222 processes unique to the CP-treated network (Fig. 4c, right), most notable was reiteration of the proteome level enrichment of vasculogenesis, cardiac development and organ regeneration absent from the untreated MI network (Fig. 4c, red). Further consistent with a reparative phenotype, the processes of transcription, morphogenesis, differentiation, and development exhibited increased prominence and significance within the CP-treated network (Fig. 4c right versus left, in pink, purple, orange, and green, respectively). This CP-induced environment had substantial reductions in metabolism and biosynthesis processes (Fig. 4c right versus left, yellow and blue circles, respectively) whereas new functions emerged. These included: protein maturation, post-translational modification and turnover; ion transport and homeostasis; as well as inflammation, immunity and stimulus responses, reflecting a possible innate response to a xenogenic immunogen despite the immunocompromised state (Fig. 4c right, gray). Thus, CP cell therapy reorganized the infarcted proteome diminishing pathogenic associations and prioritizing rejuvenative elements, traits further reinforced within the expanded network neighborhood.

### Therapy counteracted cardiac disease and adverse outcomes

**Fig. 5:**
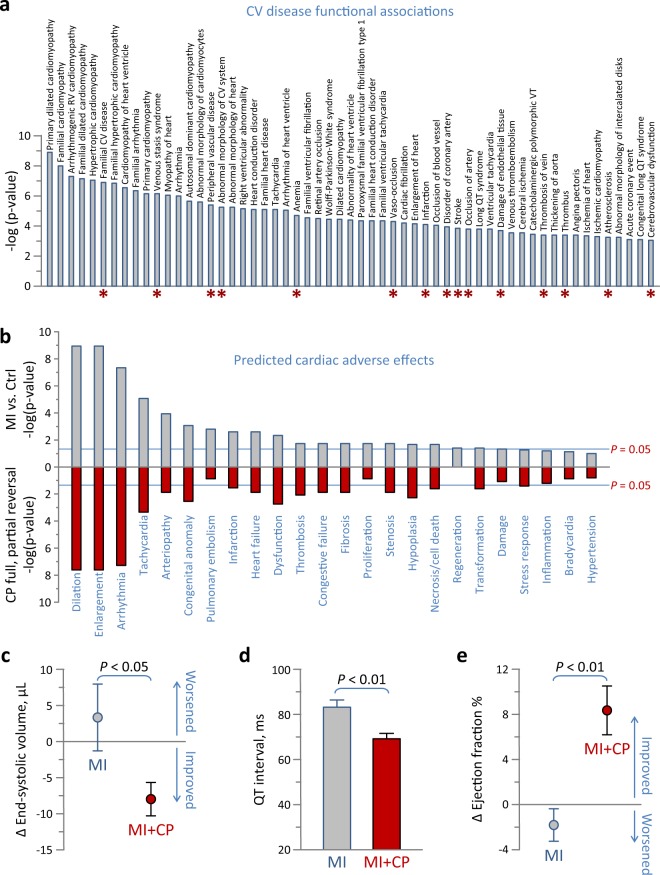
Cell therapy-reduced disease associations and -nullified adverse effects validated in vivo. **a** Ingenuity Pathway Analysis (IPA) interrogation of the infarction altered proteome for cardiovascular disease annotations revealed enriched functions (57 gray bars, *p* < 0.001), of which only 15 remained in the cell therapy altered proteome (red asterisks). **b** In IPA Tox Function analysis, hypergeometric overrepresentation predicted 24 cardiac adverse effects associated with the infarcted proteome (gray), 20 of which were significant (*p* < 0.05). Following interpretation of the reversed subproteome, nearly all adverse effects were predicted to be nullified by cell therapy (red). **c**–**e** Infarcted animals w**e**re randomized to those without (MI) versus those receiving cardiopoietic (CP) cell therapy (MI + CP), and assessed by echocardiography and electrocardiography for cardiac structure **c**, electrophysiology **d** and contractility **e**. Ischemic cardiomyopathy (MI, *n* = 8) was characterized by left ventricular dilation and pump failure, quantified by increased end-systolic volume **c** and decreased ejection fraction **e**, respectively, 4 we**e**ks post- relative to pre-randomization. In contrast, CP cell therapy (MI + CP, *n* = 16) reversed infarction induced cardiac enlargement **c** and improved performance **e**. QT prolongation, a parameter of ventricular repolarization associated with tachyarrhythmia, observed in MI (*n* = 8) was blunted in MI + CP (*n* = 8; **d**). Bars in **c**–**e** indicate standard error of the mean.

The untreated MI proteome comprised 57 ‘Cardiovascular Disease’ functional sub-annotations (Fig. 5a, gray bars), consistent with chronic pathology. Following CP cell therapy, only 15 (or 26%) remained significant (Fig. 5a, red asterisks). In fact, all cardiomyopathy-related disease features pre-eminent prior were eliminated post cell therapy, with retained associations of vaso-occlusion and vascular complications linked to the infarction insult. Furthermore, 24 predicted ‘Cardiotoxicity’ effects, prioritizing cardiac dilation and enlargement, arrhythmia, and tachycardia, and encompassing additional adverse effects associated with the failing heart characterized the MI proteome (Fig. 5b, gray). Systems interrogation of the reversed subproteome predicted that cardiac enlargement, arrhythmia/tachycardia and other salient manifestations of the failing heart respond to CP cell therapy (Fig. 5b, red). Indeed, direct echocardiographic evaluation indicated that CP cell therapy was effective in reversing infarction-induced chamber dilation. At 4 weeks post-therapy, the MI + CP cohort had reduced pathologic end-systolic size by −8 ± 2 µL (*n* = 16) contrasting with progressive increase of 3 ± 5 µL in untreated MI counterparts (*n* = 8, *p* < 0.05; Fig. 5c). QT prolongation is a risk for arrhythmia, and on electrocardiography QT interval of 83 ± 3 ms in MI (*n* = 8) was reduced to 69 ± 3 ms in MI + CP (*n* = 8, *p* < 0.01; Fig. 5d). Ultimately, the infarction-induced low ejection fraction increased by 8 ± 2% in MI + CP (*n* = 16) compared to a further decline of −2 ± 1% in MI (*n* = 8, *p* < 0.01; Fig. 5e) suggesting improved performance in vivo. Thus, the reversed subproteome was sufficient to counteract disease outcome.

### Realized trajectory from disease towards pre-disease

**Fig. 6:**
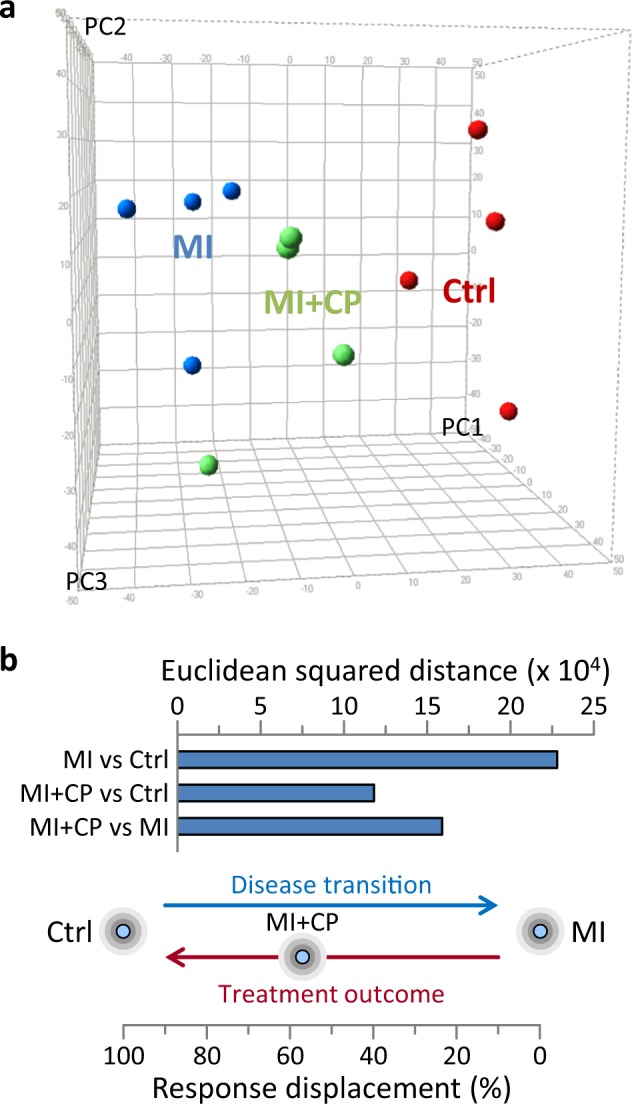
Cardiopoietic cell therapy redirects infarction modified proteome toward pre-disease state. **a** Principal component (PC) analysis of differential protein expression segregated myocardial infarction hearts (MI, blue) away from pre-disease controls (Ctrl, red), with cardiopoietic cell therapy (MI + CP, green) counterparts positioned in-between. PC1 represents 25%, PC2 13%, and PC3 11% of total data. **b** Euclidean squared distance between cohorts was maximal for MI versus Ctrl (2.28 × 10^5^) representing the full extent of disease induced transition, i.e., 100% displacement. Distance between cardiopoietic cell therapy cohort and control (MI + CP versus Ctrl) was substantially lower (1.18 × 10^5^), i.e., 52% of disease-induced displacement. Moreover, the MI + CP versus MI distance was 1.59 × 10^5^, estimating that treatment outcome (MI + CP) response displacement returned ≈57% of the way [1.18 × 10^5^/(1.18 × 10^5^ + 1.59 × 10^5^)] toward pre-disease.

Molecular signatures interpreted by spatial and distance metrics facilitate tracking of differential state space locations and transitions^23^. Aggregation of differential expression data for individual hearts and collectively for full cohorts segregated healthy controls away from infarcted counterparts, while distinguishing an intermediate position for infarcted hearts subjected to CP cell therapy (Fig. 6). Unsupervised data integration by PCA at the level of the individual, spatially positioned stem cell-treated hearts midway between maximally separated control and untreated infarcted hearts (Fig. 6a). Consistent with individual positioning, squared Euclidean distance summation of differential expression between cohorts revealed greatest separation for disease-induced transition, i.e., MI versus control (distance = 228,065). Distances between the CP cell therapy cohort and either control or MI were substantially less (at 118,470 and 159,465, respectively; Fig. 6b upper), indicating that CP cell therapy treatment returned ≈57% of the MI-induced displacement (Fig. 6b lower). Thus, in synthesis, acquired molecular data established the capacity of CP cell therapy to redirect the trajectory of an infarcted proteome back to health.

## Discussion

This systems proteomic study assessed the molecular impact of CP stem cells on the infarcted myocardium. The cardiac proteome exhibited extensive molecular remodeling imposed by chronic infarction, yet retained plasticity enabling disease course reversal by stem cell therapy. The spectrum of therapy-induced rectification comprised 2/3 of initially altered proteins. Treatment elicited proteome functional transformation, reiterated at the expanded network neighborhood level, substituting cardiomyopathic traits for reparative attributes of vasculogenesis, cardiac development and organ regeneration. Decoded molecular signatures, at systems level, positioned CP stem cell treated hearts transitioning away from disease in response to a non-stochastic resetting of the proteome.

Proteomic cartography established here a high throughput readout for protein detection, quantitation, and systems level interpretation^19,24,25^. The present compilation provides a foundational library to annotate the disease-remodeled cardiac proteome. In this regard, the measured expanse and diverse makeup of MI-provoked change extends current knowledge, focused on limited transitions measured within minutes to hours of a coronary insult^26–28^, to over 11% of the heart proteome altered 1-month post-infarction. This extent of remodeling is comparable to non-ischemic cardiomyopathy, where reported changes ranged from 9% to 12% of detected proteins^29–31^. Chronic infarction broadly impacted the myocardial proteome affecting 32 of 33 functional categories. In fact, 25 had 1 or more proteins upregulated, 29 had 1 or more downregulated, while 12 and 14 categories diverged in opposing directions. Diametric divergence of metabolic and signaling pathways initially revealed by transcriptional expression^32,33^, was reaffirmed here at proteome level, while extending the principle of reciprocity to additional categories dysregulated in opposing directions by MI. Thus, the totality of the 450 MI-altered proteins documented here effectively triples input to inform systems-based modeling, and complement existing cardiovascular resources^17,34–36^.

Proteomics is increasingly adopted in delineating intricacies of stem cell biology. While focused to date on characterizing the stem cell itself or derived active ingredients, i.e., the secretome^37–41^, the present study assessed the impact on the target of action, namely the recipient heart. Due to broad proteome derangement integral to cardiac maladaptation following infarction, a comprehensive overhaul is necessary to restore the disease-compromised molecular substrate. Traditional therapeutic strategies, such as pharmacotherapy typically hone in on a discrete pathway^42^, whereas a wider readjudication is achievable with CP stem cell-based therapy^43,44^. Indeed, as demonstrated here, CP cell treatment reduced and reversed a large proportion of proteome changes, rectifying 85% of the functional categories most affected by infarction. Non-random targeting of the compromised subproteome shares commonality with the capacity of distinct cardiac regenerative interventions to elicit a broad impact across a diseased proteome^31,45^. The pan-proteomic changes documented here detail restoration of intrinsic deficits in the cardiomyopathic heart, potentially synergistic with existing standards of care that augment cardiac performance by disrupting extrinsic neurohumoral surge imposed by sympathetic and renin/angiotensin overdrive^46^.

Here, CP cell-mediated proteome rectification eliminated nearly all detrimental functions linked to the pathology of the infarcted myocardium. In line with augmented smooth muscle and endothelial cell protein expression following CP cell transplantation post-infarction^4^, the regenerated substrate exhibited disposition towards emergence of angiogenic and endothelial development, in parallel with metabolic restitution. While a metabolic impact is traditionally observed with stem cell-mediated repair^29,30^, the prominent association with pro-vasculogenic induction distinguishes CP cell therapy. In accordance, independent reports highlight the angiogenic potential and functional neovascularization capacity of CP stem cells promoting new vessel formation in treated hearts^4,5,47^. While CP stem cell therapy did not completely restore the normal cardiac proteome, the proportion that was reversed exhibited sufficient reparative action to counteract cardiomyopathic traits and associated adverse outcomes yielding new identity. In vivo validation of structural and functional repair, corroborated by the capacity to improve cardiac performance^5^, confirmed the impact of proteome changes at organ level. In fact, the trajectory of the infarcted proteome was redirected toward the pre-infarction state.

CP cells used in the current study represent a second generation regenerative prototype^48^, one that has reached clinical phase evaluation^6–8^. Recapitulating established protocols employed in the setting of the C-CURE and CHART-1 clinical trials^6–8^, bone marrow-derived human MSCs used here were primed for cardiopoiesis using recombinant growth factors following good manufacturing practice compliant methods^4,6,7^. Progeny was characterized by increased cardiac transcription factor expression and nuclear translocation^1,4,49^. Additionally, evidence of cardiopoiesis included sarcomeric protein expression and mitochondrial ultrastructure maturation^4,7^, along with neo-vasculogenic aptitude^5^. Effectiveness with CP cell therapy was achieved regardless of whether the cardiac insult involved permanent coronary ligation, as reported previously^4^, or ischemia-reperfusion as demonstrated here. Cell therapy impact was evaluated under equivalent stem cell dosing, in randomized and blinded fashion, suggestive of consistent reparative capacity.

Notwithstanding, the current study examined a single, lineage-guided stem cell type, and thus it remains to be determined whether cell-mediated proteome restoration and functional benefit are unique to these cells or rather shared across cell-based biotherapies. Moreover, other MSC sources exist beyond bone marrow, each with distinct expression patterns and cytokine profiles^50,51^, which may yield alternative functional outcomes. Indeed, a recent proteomic study unmasked that adult bone marrow MSCs express, compared to adipose-derived MSCs, a greater yield of angiogenic proteins that translates into a more potent pro-angiogenic phenotype^41^. Furthermore, with increased understanding of the role of secretome components in mediating cell paracrine actions^52–56^, the present study delineated proteome change within the treated heart without defining the extent of contributions arising from the cell itself versus the secretome. Although various –omics approaches offer a means of addressing underlying modes of action, systems interpretation ultimately relies on probabilistic predictive tools. To increase predictive confidence, iterative analysis was conducted here at both proteome and network levels, yielding complementary support of a pro-vasculogenic transition documented in independent studies^4,5,47^, while predicted functional repair was validated experimentally by randomized multi-parametric functional and structural evaluation. Finally, while delivered stem cells, including cargo that they release, are the presumed active ingredient, confounding factors within the infarcted myocardium have been recently shown to contribute to repair, including the initial extent of disease manifestation^57^ and the pro-healing inflammatory processes^58^. Thus, the immunocompromised host may only partially recapitulate features of the regenerative response. Together, these factors warrant further consideration in the context of the present findings.

In summary, the present investigation at the recipient heart proteome level addresses the underlying molecular makeup both of changes provoked by disease and the non-aleatory rerouting towards pre-disease in response to stem cell treatment. The application of systems proteomics as demonstrated here for a clinical stage stem cell therapy thus offers a blueprint for integration of high throughput, high-resolution systems readouts to dissect and interpret complex molecular outcomes^59,60^, expanding the evidence contributing to the translational readiness of regenerative technologies.

## Methods

### Ethics approval

Animal protocols were pre-approved by the Mayo Clinic Institutional Animal Care and Use Committee, and carried out in accordance with National Institutes of Health guidelines. Procedures on living animals were performed under isoflurane anesthesia.

### Myocardial infarction and cell therapy regimen

Athymic nude male mice (2–3 months of age) underwent left anterior descending coronary artery ligation (70-min occlusion followed by reperfusion). ST elevation on the electrocardiogram confirmed myocardial infarction (MI). Four weeks post-infarction, animals were randomized at a 1:2 ratio into cohorts without (MI, *n* = 8) or with (MI + CP, *n* = 16) cell therapy. Human CP cells were generated from bone marrow-derived MSCs using an established cardiopoiesis protocol^7^. Media (15 µL), with or without CP cells (600,000 cells/heart), were epicardially delivered in infarcted left ventricles^57^.

### Protein extraction and processing

Four weeks post-therapy (i.e., 8 weeks post-infarction), age-matched control (Ctrl, *n* = 4), infarcted (MI, *n* = 4), and infarcted plus CP cell therapy (MI + CP, *n* = 4) hearts were excised and rinsed in phosphate buffered saline. Ventricles were snap frozen in liquid N_2_ and stored at −80 °C. Proteins were isolated by sequential extraction^61^, modified to maximize yield. Tissue was subjected to three rounds of mechanical homogenization by 25–30 pestle strokes at 4 °C in 150 µL of cytosolic (Cyto) extraction buffer, containing 25 mM 4-(2-hydroxyethyl)-1-piperazineethanesulfonic acid, pH 7.4, Mini-Complete™ protease inhibitor (−)EDTA cocktail (Roche Applied Science, Indianapolis, IN), and 1% phosphatase inhibitor cocktails 2 and 3 (Sigma, St. Louis, MO). Homogenates were centrifuged 5 min at 16,000×*g*, with supernatant pooled as the ‘Cyto’ extract. Remaining pellet was subjected to three rounds of homogenization at 4 °C in 150 µL of membrane, myofilament, and organelle (MMO) extraction buffer, consisting of 7 M urea, 2 M thiourea, and 2% 3-((3-cholamidopropyl) dimethylammonio)-1-propanesulfonic acid, and centrifuged 5 min at 16,000×*g* with supernatant pooled as the ‘MMO’ extract. Extracts were quantified in triplicate by Bio-Rad protein assay (Hercules, CA) using the microassay procedure with bovine γ-globulin standard. Samples (30 µg) were resolved by 10.5–14% gradient Criterion Tris–HCl precast (Bio-Rad) sodium dodecyl sulfate–polyacrylamide gel electrophoresis, using separate gels for ‘Cyto’ and ‘MMO’ extracts, and stained with Coomassie brilliant blue R-250. Gels were processed for mass spectrometry by segmenting extract lanes to reduce complexity of each segment for individual mass spectrometry runs.

### Peptide spectral acquisition by nanoelectrospray mass spectrometry

Resolved gel sections were de-stained and prepared for mass spectrometry by reduction, alkylation, and tryptic digestion, followed by peptide extraction. Dried peptides were re-suspended in 0.2% formic acid, 0.1% trifluoroacetic acid, and 0.002% zwittergent 3–16 (Calbiochem, San Diego, CA). Samples were analyzed by nano-flow liquid chromatography electrospray tandem mass spectrometry (nanoLC–ESI–MS/MS) using a Q-Exactive Hybrid Quadrupole Orbitrap mass spectrometer (Thermo Fisher Scientific, Bremen, Germany) coupled to a Thermo UltiMate 3000 RSLCnano HPLC system. Sample run order, randomized to negate chromatographic bias, alternated with 30 min blank runs to avoid carry-over. Peptides were loaded onto a 250 nL OPTI-PAK trap (Optimize Technologies, Oregon City, OR) packed with Michrom Magic C8, 5 µm solid phase (Michrom Bioresources, Auburn, CA). Chromatography was performed using 0.2% formic acid in solvents A (98% water, 2% acetonitrile) and B (80% acetonitrile, 10% isopropanol, 10% water), over a 2–45% B gradient for 60 min at 400 nL/min through a 100 µm × 35 cm PicoFrit column (New Objective, Woburn, MA) hand-packed with Agilent Poroshell 120 EC-C18 (Agilent Scientific Instruments, Santa Clara, CA). MS1 survey scans were acquired from 350 to 2000*m*/*z* at resolution 70,000 (at 200*m*/*z*) with an automatic gain control target of 3 × 10^6^ ions and 60-ms maximum ion inject time, followed by high-energy collisional dissociation MS/MS on the top 15 ions at resolution 17,500 targeting 2 × 10^5^ ions with 60-ms maximum ion inject time. Dynamic exclusion placed selected ions on an exclusion list for 60 s.

### Mass spectrometry data analysis

A total of 228 raw MS/MS files were acquired for 12 biological samples (*n* = 4 per experimental group), consisting of 19 separate MS/MS fractions per sample. Raw files were processed in MaxQuant v1.5.1.2^62^, using the Andromeda search engine for multidimensional label-free quantification (LFQ) based on extracted ion chromatogram with applied fastLFQ settings, to enable relative quantification across cohorts. Spectra were searched against the UniProt mouse database, combining forward and reverse peptides as decoys to estimate false discovery rate (FDR), calculated as proportion of reverse relative to forward plus reverse sequence matches. Calculated FDR was 0.28%. MS/MS search parameters included trypsin/P digestion, with fixed modification of cysteine carbamidomethylation, and variable modifications of amino-terminal protein acetylation, glutamate to pyro-glutamate modification and methionine oxidation. Maximum charge was +7, with up to three dynamic modifications, three missed cleavages, and a minimum of 7 amino acids. Mass tolerance was set at 20 ppm for first search and 10.5 ppm for main search, with isotope mass tolerance of 2 ppm. LFQ included spectral features matching a theoretical MS/MS peptide spectrum, with peptide identification maximized by MaxQuant’s ‘Match Across Groups’ feature assigning spectral features from one MS/MS run to corresponding aligned masses and retention times detected in other runs. Identified peptides were rolled into protein assignments, with matches requiring a minimum of two peptides. The mass spectrometry data were deposited to the ProteomeXchange Consortium via the PRIDE^63^ partner repository with the dataset identifier PXD017381.

### Cohort differential expression

Protein differential expression was calculated using R. Protein spectral intensities were normalized using total ion current, followed by log_2_ transformation to generate normal distributions. This was iterated for each protein, with data fit to a generalized linear model of counts versus groups assuming Gaussian distribution. Protein levels were compared by two-sided ANOVA, with Gaussian linked function, using a minimum two-fold change, *p* < 0.01 considered differentially expressed. Agglomerative heatmaps and principal component analysis (PCA) were conducted with ClustVis (https://biit.cs.ut.ee/clustvis_large/)^64^, with heatmaps visualized using ClustVis and PCA data exported to Spotfire (TIBCO, Palo Alto, CA) for 3-D visualization. Experimental group spatial separation was calculated by Euclidean squared distance, the sum of squared fold change differences for all proteins identified as differentially expressed.

### Pathway and network informatics

Differentially expressed proteins were submitted to Ingenuity Pathway Analysis (IPA, QIAGEN Bioinformatics, Hilden, Germany) for pathway and network analysis. Disease and cell therapy-associated networks (Networks tab), upstream regulators of proteome changes (Upstream Analysis tab, Upstream Regulators sub-heading), and enriched functional annotations (Diseases & Functions tab) associated with ‘Cardiovascular System Development and Function’ and ‘Cardiovascular Disease’ (Diseases and Bio Functions sub-heading) and cardiac adverse effects (‘Cardiotoxicity’ categories under the Tox Functions sub-heading) were derived, using Fisher’s exact test for *p*-value calculations. Disease-induced differential expression and response to therapy were collectively evaluated at network level by merging IPA-predicted node relationships into composite networks. Network relationships were exported for visualization and interrogation using Cytoscape (v. 3.7.1)^65^. Networks were characterized as an undirected network using NetworkAnalyzer to generate degree distributions and evaluate network topology^66^. The Cytoscape application ‘BiNGO’ (Biological Network Gene Ontology) defined Gene Ontology (GO) Biological Process enrichment for each network using hypergeometric distribution with Benjamini–Hochberg FDR correction for *p*-value calculation, enabling distinction of processes unique to disease or cell therapy^67^.

### Echocardiography and electrocardiography

Ejection fraction was quantified in blinded fashion by echocardiography (30 MHz transducer; Vevo3100 with MX400, Vevo2100 with MS400, FUJIFILM VisualSonics, Toronto, Canada), as the difference between end-diastolic and end-systolic volumes divided by end-diastolic volume^68^. Arrhythmic risk was assessed by QT interval on electrocardiograms (LabChart 7, AD Instruments). Nonparametric Mann–Whitney *U* test evaluated significance between cohorts (JMP Pro 14.1.0, SAS Institute Inc., Cary, NC). Data are presented as mean ± SEM. A *p*-value < 0.05 was considered significant.

### Reporting summary

Further information on research design is available in the Nature Research Reporting Summary linked to this article.

## Supplementary information

Supplementary Data Set and Figure Legends

Supplementary Data Set 1

Supplementary Data Set 2

Reporting Summary

## Data Availability

The authors declare that all data supporting the findings of this study are available within the article and its supplementary material files, or from the corresponding author upon reasonable request. Proteomic mass spectrometry data are available via the public repository ProteomeXchange with identifier PXD017381.
