# Supplementary material for: Cardiopoietic stem cell therapy restores infarction-altered cardiac proteome
Source: NPJ Regen Med. 2020 Mar 12;5:5. doi: 10.1038/s41536-020-0091-6 (PMC7067830; doi:10.1038/s41536-020-0091-6)
Supplement: Supplementary file 1 — Supplementary Data Set and Figure Legends [file 41536_2020_91_MOESM1_ESM.pdf]

Arrell et al.

Cardiopoietic Stem Cell Therapy Restores Infarction-Altered Cardiac Proteome

Supplementary Data

Supplementary Data Set 1. Peptides identified by tandem mass spectrometry. Total assigned peptides, contaminant peptides, reverse sequence peptides, and full peptide lists are presented.

Supplementary Data Set 2. Protein assignment based on peptide identities. Proteins are listed with peptide, gene ontology, and functional cluster associations.

Supplementary Data Figure 1. **Inter- and intra-group reproducibility in protein signal intensity with narrow variance ensuring reliable cross group comparison.** (**a**) Rank ordered signal intensity for all 3,987 assigned proteins was superimposed, demonstrating comparable spectral abundance across experimental cohorts, i.e., control (Ctrl), myocardial infarction (MI), and MI plus cardiopoietic cell therapy (MI+CP). Under each condition, protein signal corresponds to mean intensity of four biological replicates. (**b**) Intra-group coefficient of variance (CV) was consistently low across conditions (Ctrl, MI, MI+CP), indicated by quintile measurements with ≈1% median inter-sample variability, and ≈3.6% variability at the 90th percentile.

Supplementary Data Figure 2. Infarction- and cardiopoietic cell therapy-dependent proteome networks exhibit partial overlap. (**a**) Merged interactions identified by Ingenuity Pathway Analysis (IPA) for the infarction altered proteome (MI vs. Ctrl, –CP) resulted in a 768 node network comprising 9,974 connections (edges), with degree distribution [X(*k*) *versus* *k*] exhibiting scale-free topology, where an

extremely small number of highly connected nodes serve as hubs (graph). (**b**) IPA network generation from the cell therapy altered proteome (MI+CP vs. Ctrl, +CP) generated a 578 node network consisting of 8,808 edges and scale-free topology (graph), with partial overlap between –CP and +CP networks (inset). Nodes common to both networks are colored yellow, while nodes unique to an individual network are blue. Red lines indicate power law trends with equations and correlations in red text. Computed topological properties plotted are node degree (*k*), number of edges connected to each node, *versus* number of nodes with degree *k*, or X(*k*).

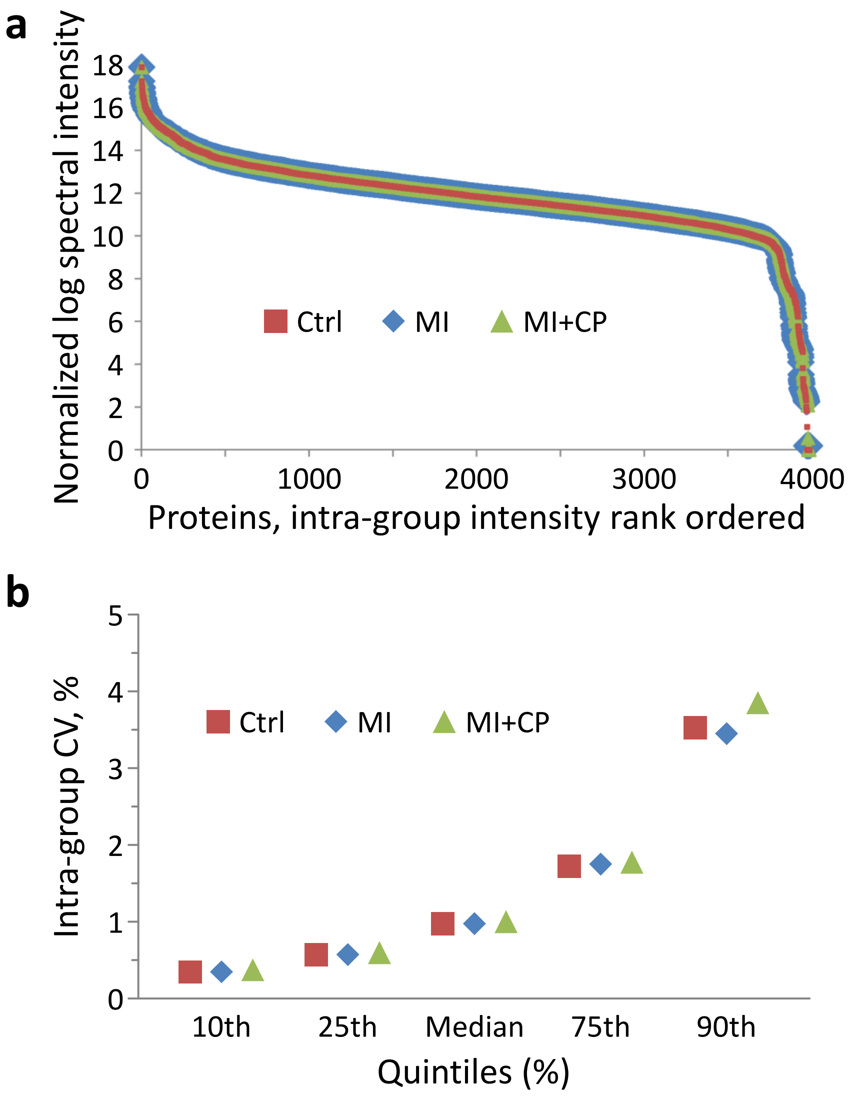

Supplementary Data Figure 1

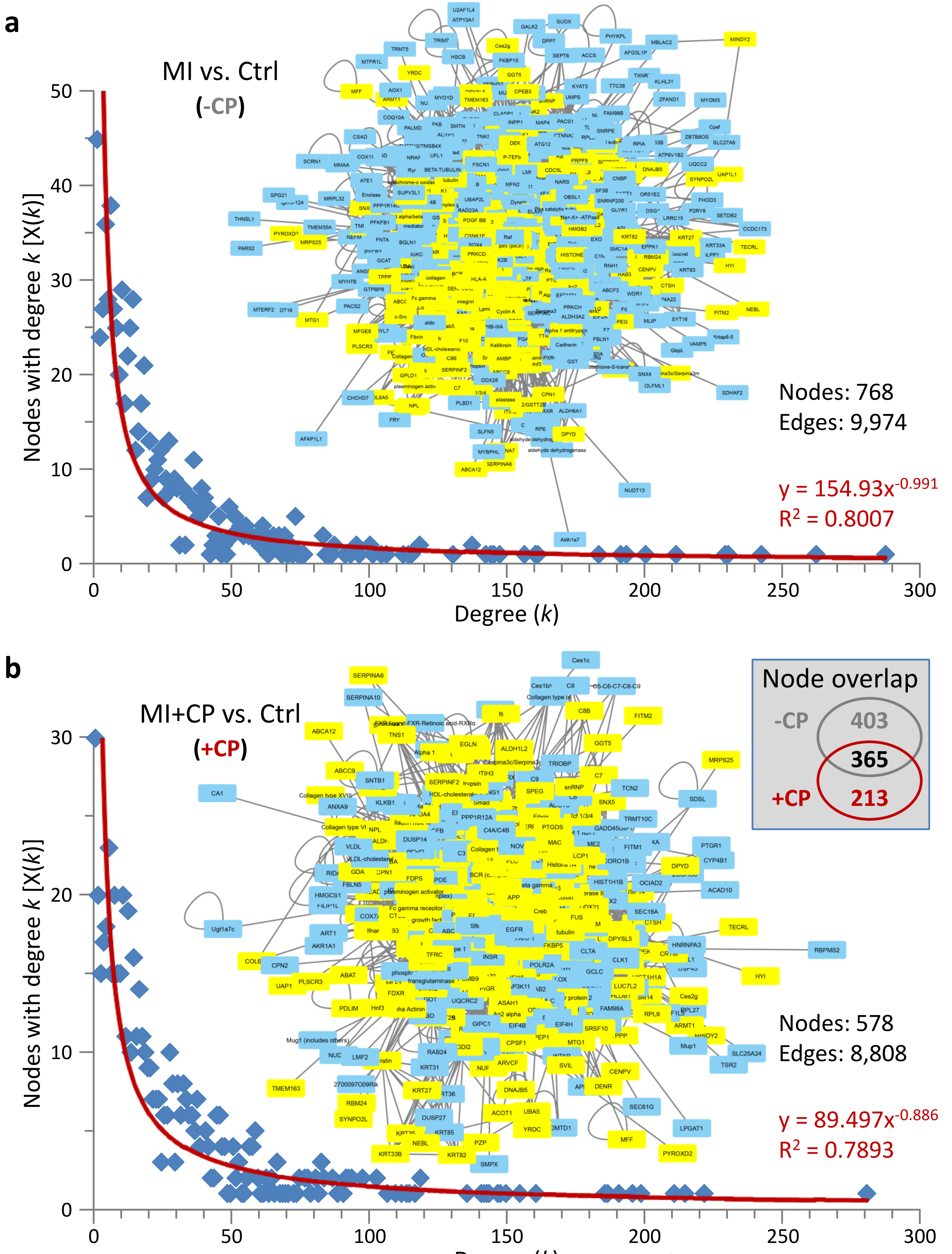

Supplementary Data Figure 2
